# The Hanging Strap Method: A Safe and Easy-to-Use Surgical Technique for Surgeons-in-Training Performing Difficult Laparoscopic Cholecystectomy

**DOI:** 10.7759/cureus.66739

**Published:** 2024-08-12

**Authors:** Kei Harada, Ippei Yamana, Yusuke Uemoto, Yuichiro Kawamura, Takahisa Fujikawa

**Affiliations:** 1 Surgery, Kokura Memorial Hospital, Kitakyushu, JPN

**Keywords:** critical view of safety, acute cholecystitis, bile duct injury, surgeons-in-training, difficult laparoscopic cholecystectomy

## Abstract

Introduction

Surgeons-in-training (SIT) perform laparoscopic cholecystectomy (LC); however, it is challenging to complete the procedure safely in difficult cases. We present a surgical technique during difficult LC, which we named the hanging strap method.

Methods

We retrospectively compared the perioperative outcomes between patients undergoing difficult LC with the hanging strap method (HANGS, n = 34), and patients undergoing difficult LC without the hanging strap method (non-HANGS, n = 56) from 2022 and 2024. Difficult LC was defined as cases classified as more than grade II cholecystitis by the Tokyo Guidelines 18 and cases when LC was undergoing over five days after the onset of cholecystitis.

Results

The proportion of SIT with post-graduate year (PGY) ≤ 7 was significantly higher in the HANGS group than in the non-HANGS group (82.4% vs. 33.9%, P < 0.001). The overall rate of bile duct injury (BDI), postoperative bile leakage and operative mortality were zero in the whole cohort. There were no significant differences between the HANGS and non-HANGS groups in background characteristics, operative time (122 min vs. 132 min, P = 0.830) and surgical blood loss (14 mL vs. 24 mL, P = 0.533).

Conclusions

Our findings suggested that the hanging strap method is safe and easy to use for difficult LC. We recommend that the current method be selected as one of the surgical techniques for SIT when performing difficult LC.

## Introduction

Although laparoscopic cholecystectomy (LC) is generally a safe procedure for gallbladder-related diseases, in cases of cholecystitis with severe inflammation, it should be performed with caution to avoid serious complications such as bile duct injury (BDI) [1,2]. It has been reported that BDI is particularly likely to occur in difficult LC for chronic cholecystitis and cholecystitis obstruction due to gallbladder neck stones [3]. According to several reports, the incidence of BDI with LC is between 0.3% and 0.42%, but it could be as high as 3% in difficult LC [4,5].

To prevent complications such as BDI during LC, recognition of important anatomy including the critical view of safety (CVS) and various surgical techniques have been proposed [6,7]. Although strategies to avoid these complications are considered useful, difficult LC remains a challenging procedure and complications still occur. LC is a safe and feasible procedure for surgeons-in-training (SIT) to learn LC. Furthermore, many studies have shown that surgical outcomes are comparable to those of trained surgeons [8]. However, there are reports when SIT performs the difficult LC, the conversion rate to open surgery is higher than that of trained surgeons, leading to increased complications [9].

Herein, we present a surgical technique during difficult LC, which we named the hanging strap method, and assess the safety and feasibility of the current technique with special reference to the operations performed by SIT.

## Materials and methods

We investigated the prospectively collected surgery database of a single institution for relevant cases from April 2022 to April 2024, and 238 patients diagnosed with symptomatic cholelithiasis, acute cholecystitis, chronic cholecystitis, and preventive surgeries for the common bile duct stones and gallbladder polyps underwent LC. Among them, excluding patients in which surgery was changed to subtotal cholecystectomy or open surgery, and undergoing non-difficult LC, 90 consecutive patients undergoing difficult LC were enrolled in the current cohort. Difficult LC was defined as cases classified as more than grade II cholecystitis by the Tokyo Guidelines 18 [5] and cases when LC was undergoing at least five days after the onset of cholecystitis. The patients were divided into two groups: patients undergoing difficult LC with the hanging strap method (HANGS, n = 34), and patients undergoing difficult LC without the hanging strap method (non-HANGS, n = 56).

All procedures were conducted by a board-certified attending surgeon or SIT with post-graduate year (PGY) ≤ 7 under supervision at our institution. In Japan, gastroenterological surgeon specialists are generally certificated seven years after graduation, so we defined SIT as those with PGY ≤ 7. In both groups, SIT performed LC under the supervision of the attending surgeon and completed the procedure without any change of surgeon in all cases. The American Society of Anesthesiologists Physical Status (ASA PS) classification is used to assess a patient's preoperative physical health status and risk for perioperative complications. The primary surgical outcomes were determined by surgical blood loss, duration of operation time, the presence of BDI, operative mortality, and postoperative bile leakage. Operative mortality is defined as the occurrence of death during a period of 30 days following a surgical procedure.

The Kokura Memorial Hospital Clinical Research Ethics Committee authorized the protocol of the current study (#24062102), which complied with the Declaration of Helsinki.

Statistical analysis

Continuous variables were expressed as medians with ranges. Statistical comparisons were performed using the chi-square test or Fisher's exact test, and Student's t-test was used to analyze differences between continuous values. All P-values were two-sided, and P-values < 0.05 were considered statistically significant. All statistical analyses were performed using EZR (Saitama Medical Center, Jichi Medical University, Saitama, Japan), a graphical user interface for R (The R Foundation for Statistical Computing, Vienna, Austria, version 1.67) [10].

LC procedure

LC was typically performed with four ports. The ports were placed following the American approach [11]. The undulation was set to a pneumoperitoneum pressure of 8 mmHg with CO2. The operator's 5-mm working port (for the operator's right hand) was inserted at the epigastric lesion. In our hospital, if LC is expected to be difficult, the operator may change the 5-mm port for the upper abdominal lesion to a 12-mm port. In addition, an additional port may be inserted so that the assistant surgeon can use both hands during LC. A 5-mm port for the operator's left hand was inserted at the right subcostal area along the right mid-clavicular line. A 5- or 10-mm flexible video scope was inserted through the 12-mm port that was placed at the umbilicus. For gallbladder retraction, a 5-mm port was placed at the subcostal area along the anterior axillary line. Under pneumoperitoneum, first, identify the hepatic hilum if possible. After dissecting the cholecystitis-related adhesions around the gallbladder, surface landmarks such as the Rouvière's sulcus, the segment IV of the liver, the infundibulum of the gallbladder, and the bile duct are identified. At our institution, the gallbladder serosal incision begins above the line connecting the Rouvière's sulcus and the base of segment IV of the liver. After that, we use the hanging strap method in difficult LC for cases with severe inflammation. After recognizing as much CVS as possible, the cystic duct is then clipped and divided. We prefer to clip the cystic artery to avoid bleeding during dissection. The attached gallbladder is then dissected from the liver and extracted.

Surgical technique of the hanging strap method

**Figure 1:**
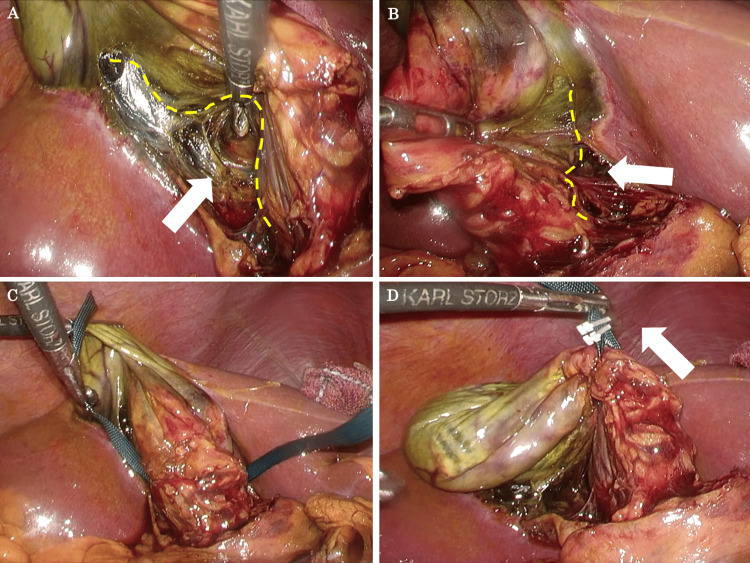
Steps for the hanging strap method (A) With medial and superior traction of the gallbladder, the avascular plane between the posterior cystic artery and the posterior gallbladder wall (yellow dotted line) is dissected. (B) With lateral and superior traction of the gallbladder, the posterior gallbladder wall (yellow dotted line) above the lymph node is dissected. (C) A polyester tape is passed through the space behind the liver bed and the gallbladder body. (D) The polyester tape is clipped and the assistant lifts it up.

After incising the gallbladder serosal membrane bilaterally down to the fundus of the gallbladder, dissection of the gallbladder body is started. Once the gallbladder body is completely dissected from the liver bed, the gallbladder is lifted using 5-mm wide green polyester tape (NITCHO Corporation, Japan). The gallbladder is lifted by attaching a clip to polyester tape so that the assistant holds it. The procedure for the hanging strap method has been described above and is a very easy-to-use surgical technique. The complete procedure of the hanging strap method for difficult LC is shown in Figure 1 and Video 1. If the scarring of the gallbladder wall due to cholecystitis is not severe, it is usually not difficult to dissect the posterior wall of the gallbladder body from the liver bed. If the dorsal first dissection of the gallbladder body is extremely difficult, the procedure is changed to the dome-down method [12]. This procedure makes the gallbladder lifted up on the polyester tape look like a hanging strap, so we named this surgical technique the hanging strap method.

**Video 1:** Surgical technique for the hanging strap method. Demonstration of two cases.

## Results

Background characteristics of the HANGS and non-HANGS group are listed in Table 1 and surgical outcomes are listed in Table 2. The proportion of SIT with PGY ≤ 7 was significantly higher in the HANGS group than in the non-HANGS group (82.4% vs. 33.9%, P < 0.001). The proportion of males is higher than in the non-HANGS group than in the HANGS group (76.8% vs. 50.0%, P < 0.012). There were also similarities between the HANGS group and non-HANGS group in the mean age (72 years vs. 71 years, P = 0.873), BMI (25.64 kg/m^2^ vs. 24.12 kg/m^2^, P = 0.148), poor ASA PS (32.4% vs. 33.9%, P = 1.000), emergency surgery (64.7% vs. 60.7%, P = 0.823). The overall rate of BDI, postoperative bile leakage and operative mortality were zero in the whole cohort. There were no significant differences between the HANGS and non-HANGS groups in background characteristics, operative time (122 min vs. 132 min, P = 0.830), and surgical blood loss (14 mL vs. 24 mL, P = 0.533).

**Table 1:** Background characteristics and surgical outcomes in the current cohort BMI: body mass index; ASA PS Classification: American Society of Anesthesiologists Physical Status Classification; PGY: post-graduate year; HANGS: patients undergoing difficult LC with the hanging strap method; non-HANGS: patients undergoing difficult LC without the hanging strap method

Variables	HANGS group (n=34)	Non-HANGS group (n=56)	P-value
Age, y, median (range)	72 (39-90)	71 (40-94)	0.873
Male gender, n (%)	17 (50.0)	43 (76.8)	0.012
BMI, kg/m^2^, median (range)	25.6 (20.2-35.2)	24.1 (16.9-36.1)	0.148
ASA PS Classification 3 or higher, n (%)	11 (32.4)	19 (33.9)	1.000
Emergency surgery, n (%)	22 (64.7)	34 (60.7)	0.823
PGY ≤ 7, n (%)	28 (82.4)	19 (33.9)	<0.001

**Table 2:** Surgical outcomes in the current cohort HANGS: patients undergoing difficult LC with the hanging strap method; non-HANGS: patients undergoing difficult LC without the hanging strap method

Variables	HANGS group (n=34)	Non-HANGS group (n=56)	P-value
Surgical blood loss, mL, median (range)	14 (5-405)	24 (4-760)	0.533
Duration of operation time, min, median (range)	122 (72-249)	132 (43-248)	0.830
Postoperative bile leakage, n (%)	0 (0.0)	0 (0.0)	N/A
Bile duct injury during operation, n (%)	0 (0.0)	0 (0.0)	N/A
Operative mortality, n (%)	0 (0.0)	0 (0.0)	N/A

## Discussion

LC has become a safe standard surgical procedure for gallbladder-related diseases in recent years, and that is often performed by young SIT as training in gastrointestinal laparoscopic surgery [9,13,14]. The difficulty of LC procedures ranges from easy to difficult, especially that is much more difficult in cases of cholecystitis with severe inflammation such as TG18 [5] grade 2 or higher, cases in which more than a few days have passed since the onset of symptoms, and cases caused by obstruction by stones in the neck of the gallbladder [15,16]. During LC, BDI is a major complication with technical errors and misidentification of the anatomy being the main risk factors [17,18]. Several reports suggest that the incidence of BDI with LC is 0.3-0.42%, but may be as high as 3% in the case of difficult LC with chronic cholecystitis accompanied by atrophy and severe inflammation [4,19]. The concept of CVS has been adopted around the world to prevent this misidentification of the anatomy [20]. Although there are other various strategies and initiatives, BDI during LC remains a common occurrence, particularly in cases with severe inflammation [21,22].

Our study reports the effect of the hanging strap method on difficult LC performed by SIT. The hanging strap method starts with the dorsal dissection of the gallbladder body first. The effectiveness of this dorsal dissection of the gallbladder body has been suggested in various papers, and we believe it to be an effective surgical technique [23,24]. It is important for this procedure to concentrate on dissection along the gallbladder wall in order to prevent damage to the liver parenchyma and the peripheral branches of the middle hepatic vein [25,26]. After the procedure, once the gallbladder body has been completely detached from the liver bed, it is lifted with polyester tape. There are three reasons to use polyester tape. The first reason is reduced tissue damage: polyester tape’s smooth, non-abrasive surface minimizes the risk of damage to the gallbladder lining. Second, it is easy to handle. Polyester tape's predictable mechanical properties make it easy to handle and manipulate, even in laparoscopic surgery. Third, it has excellent traction and stability. Polyester tape's high tensile strength allows it to firmly grip the gallbladder without slipping, which is essential for precise manipulation during LC.

**Figure 2:**
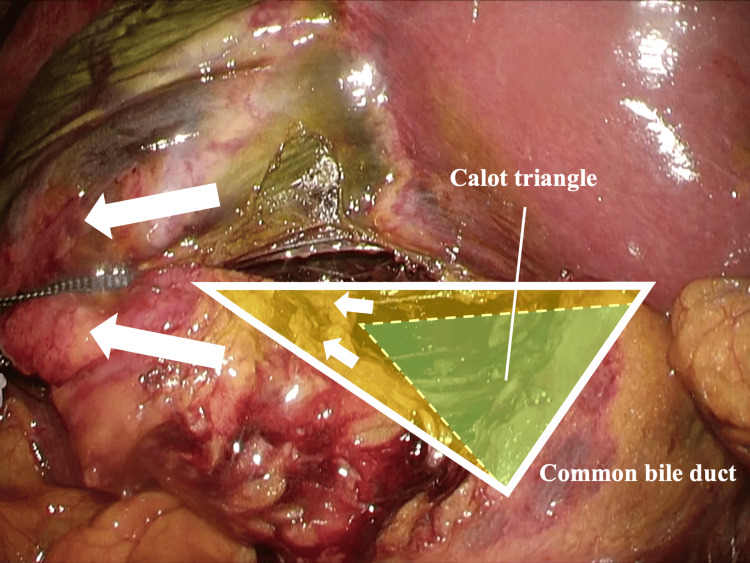
Opening of the Calot's triangle during the hanging strap method The green triangular area represents a Calot's triangle. An assistant traction by using the hanging strap method can effectively open the Calot's triangle (yellow area).

Based on our study, we considered that the hanging strap method has three effects. First, it effectively opens the Calot's triangle (Figure 2). According to several papers, in addition to the concept of CVS, effective traction of the gallbladder and the recognition of the Calot's triangle region as important landmarks are particularly important to prevent BDI [18,23,27]. However, in cases of cholecystitis with significant gallbladder enlargement, sufficient gallbladder traction is difficult, and in cases with gallbladder wall thickening and severe fibrosis, it is difficult to effectively open the Calot's triangle [27]. Traction from the gallbladder body to the gallbladder neck with polyester tape can effectively open the Calot's triangle, helping to avoid injuring important structures.

Second, it can prevent gallbladder and liver parenchyma injury caused by traction by an assistant. Having an assistant continue to grasp the gallbladder with laparoscopic forceps can lead to gallbladder perforation. Intraoperative gallbladder perforation with spillage of bile and gallstones during LC is associated with an increased risk of developing intra-abdominal abscesses postoperatively [28,29]. Therefore, it is extremely useful to be able to reduce the risk of gallbladder perforation caused by having an assistant forceps pull on the gallbladder, which is difficult to grasp due to its fragility or enlargement. In addition, when it is difficult to pull up the gallbladder, the assistant may use forceps to compress and lift the liver parenchyma, but this can result in force being applied to a single point, which can easily lead to damage to the liver parenchyma. The hanging strap method allows force to be applied over a wide area rather than just a single point, preventing damage to the liver parenchyma.

Third, it can be easily performed even by SIT. In our study, all difficult LCs were conducted by a board-certified attending surgeon or SIT with PGY ≤ 7 under supervision at our institution. The operator completed the procedure without any change of surgeon in all cases. The proportion of SIT with PGY ≤ 7 was significantly higher in the HANGS group than in the non-HANGS group (82.4% vs. 33.9%, P < 0.001). There were no significant differences between the HANGS and non-HANGS groups in background characteristics, operative time (122 min vs. 132 min, P = 0.830) and surgical blood loss (14 mL vs. 24 mL, P = 0.533). In other words, these results indicate that even SIT can reproduce satisfactory outcomes using the current procedure. From the perspective of surgical technique, the body of the gallbladder can be easily detached from the liver bed unless severe fibrosis prevents the layer from being maintained. In addition, passing the polyester tape through the space between the liver bed and the detached gallbladder is an easy procedure. Since appropriate tension is applied to the Calot's triangle, the surgeon's non-dominant hand is free, making it easier to perform subsequent surgical procedures.

The hanging strap method is not a surgical technique to force total cholecystectomy in LC with a high risk of BDI. Ultimately, in addition to conventional strategies, we believe that bailout surgery (e.g., change to open surgery or partial resection) is appropriate when various factors that make total cholecystectomy difficult are identified or can’t be performed the hanging strap method [5,30].

The current investigation has some limitations. First, the study was retrospective, limiting its ability to analyze the surgical technique effects. Second, the sample size was relatively small. Increasing the sample size will help make the suggestions more reliable. We believe that the hanging strap method is a positive step toward effectively combating difficult LC experienced by SIT.

## Conclusions

Our findings indicate that even SIT can reproduce satisfactory outcomes using the hanging strap method. Also, the use of the current method may be expected to reduce BDI and improve the completion rate of SIT. We suggested that the hanging strap method is safe and easy to use for difficult LC, and recommend that the current method be selected as one of the surgical techniques for SIT when performing difficult LC.

## References

[1] Borowicz MR, Adams DB, Simpson JP, Cunningham JT (1995). Management of biliary strictures due to laparoscopic cholecystectomy. J Surg Res.

[2] Strasberg SM, Sanabria JR, Clavien PA (1992). Complications of laparoscopic cholecystectomy. Can J Surg.

[3] Richardson AJ, Brancatisano R, Avramovic J, Roney W, Little JM (2024). Injuries to the bile duct resulting from laparoscopic cholecystectomy. Aust N Z J Surg.

[4] Krähenbühl L, Sclabas G, Wente MN, Schäfer M, Schlumpf R, Büchler MW (2001). Incidence, risk factors, and prevention of biliary tract injuries during laparoscopic cholecystectomy in Switzerland. World J Surg.

[5] Wakabayashi G, Iwashita Y, Hibi T (2018). Tokyo Guidelines 2018: surgical management of acute cholecystitis: safe steps in laparoscopic cholecystectomy for acute cholecystitis (with videos). J Hepatobiliary Pancreat Sci.

[6] van de Graaf FW, Zaïmi I, Stassen LP, Lange JF (2018). Safe laparoscopic cholecystectomy: a systematic review of bile duct injury prevention. Int J Surg.

[7] Buddingh KT, Nieuwenhuijs VB, van Buuren L, Hulscher JB, de Jong JS, van Dam GM (2011). Intraoperative assessment of biliary anatomy for prevention of bile duct injury: a review of current and future patient safety interventions. Surg Endosc.

[8] Atabek U, Spence RK, Pello MJ, Alexander JB, Villanueva D, Camishion RC (1993). Safety of teaching laparoscopic cholecystectomy to surgical residents. J Laparoendosc Surg.

[9] Böckler D, Geoghegan J, Klein M, Quasim W, Turan M, Meyer L, Scheele J (1999). Implications of laparoscopic cholecystectomy for surgical residency training. JSLS.

[10] Kanda Y (2013). Investigation of the freely available easy-to-use software 'EZR' for medical statistics. Bone Marrow Transplant.

[11] Carlomagno N, Santangelo M, Romagnuolo G, Antropoli C, La Tessa C, Renda A (2014). Laparoscopic cholecystectomy: technical compromise between French and American approach. Presentation of an original technique. Ann Ital Chir.

[12] Rosenberg J, Leinskold T (2004). Dome down laparosonic cholecystectomy. Scand J Surg.

[13] Overby DW, Apelgren KN, Richardson W, Fanelli R (2010). SAGES guidelines for the clinical application of laparoscopic biliary tract surgery. Surg Endosc.

[14] Deziel DJ, Millikan KW, Staren ED, Doolas A, Economou SG (1993). The impact of laparoscopic cholecystectomy on the operative experience of surgical residents. Surg Endosc.

[15] Inoue K, Ueno T, Douchi D (2017). Risk factors for difficulty of laparoscopic cholecystectomy in grade II acute cholecystitis according to the Tokyo guidelines 2013. BMC Surg.

[16] Nidoni R, Udachan TV, Sasnur P, Baloorkar R, Sindgikar V, Narasangi B (2015). Predicting difficult laparoscopic cholecystectomy based on clinicoradiological assessment. J Clin Diagn Res.

[17] Hunter JG (1991). Avoidance of bile duct injury during laparoscopic cholecystectomy. Am J Surg.

[18] Iwashita Y, Hibi T, Ohyama T (2017). Delphi consensus on bile duct injuries during laparoscopic cholecystectomy: an evolutionary cul-de-sac or the birth pangs of a new technical framework?. J Hepatobiliary Pancreat Sci.

[19] Nuzzo G, Giuliante F, Giovannini I (2005). Bile duct injury during laparoscopic cholecystectomy: results of an Italian national survey on 56 591 cholecystectomies. Arch Surg.

[20] Strasberg SM, Hertl M, Soper NJ (1995). An analysis of the problem of biliary injury during laparoscopic cholecystectomy. J Am Coll Surg.

[21] Schwaitzberg SD, Scott DJ, Jones DB, McKinley SK, Castrillion J, Hunter TD, Michael Brunt L (2014). Threefold increased bile duct injury rate is associated with less surgeon experience in an insurance claims database: more rigorous training in biliary surgery may be needed. Surg Endosc.

[22] Georgiades CP, Mavromatis TN, Kourlaba GC (2008). Is inflammation a significant predictor of bile duct injury during laparoscopic cholecystectomy?. Surg Endosc.

[23] Iskandar M, Fingerhut A, Ferzli G (2024). Posterior infundibular dissection: safety first in laparoscopic cholecystectomy. Surg Endosc.

[24] Song J, Chen J, Zheng S (2022). Lateral dorsal infundibular approach: an alternative option for the safe completion of difficult laparoscopic cholecystectomy. BMC Surg.

[25] Pesce A, Fabbri N, Feo CV (2023). Vascular injury during laparoscopic cholecystectomy: an often-overlooked complication. World J Gastrointest Surg.

[26] Ball CG, MacLean AR, Kirkpatrick AW, Bathe OF, Sutherland F, Debru E, Dixon E (2006). Hepatic vein injury during laparoscopic cholecystectomy: the unappreciated proximity of the middle hepatic vein to the gallbladder bed. J Gastrointest Surg.

[27] Sekimoto M, Tomita N, Tamura S, Ohsato H, Monden M (1998). New retraction technique to allow better visualization of Calot's triangle during laparoscopic cholecystectomy. Surg Endosc.

[28] Rice DC, Memon MA, Jamison RL (1997). Long-term consequences of intraoperative spillage of bile and gallstones during laparoscopic cholecystectomy. J Gastrointest Surg.

[29] Nagata K, Fujikawa T, Oka S, Osaki T (2022). A case of intractable lung abscess following dropped gallstone-induced subphrenic abscess: a rare postoperative complication caused by dropped gallstone during laparoscopic cholecystectomy. Cureus.

[30] Ledezma Dominguez J, Tariq N, Martins RS, Jawad G, Fisher AD, Maqbool B (2024). Bailout surgery for difficult gallbladders: surgical approach and outcomes. Am Surg.

